# Shared Resistance to Aging and ALS in Neuromuscular Junctions of Specific Muscles

**DOI:** 10.1371/journal.pone.0034640

**Published:** 2012-04-02

**Authors:** Gregorio Valdez, Juan C. Tapia, Jeff W. Lichtman, Michael A. Fox, Joshua R. Sanes

**Affiliations:** 1 Department of Molecular and Cellular Biology and Center for Brain Science, Harvard University, Cambridge, Massachusetts, United States of America; 2 Department of Anatomy and Neurobiology, Virginia Commonwealth University, Richmond, Virginia, United States of America; University of Edinburgh, United Kingdom

## Abstract

Normal aging and neurodegenerative diseases both lead to structural and functional alterations in synapses. Comparison of synapses that are generally similar but respond differently to insults could provide the basis for discovering mechanisms that underlie susceptibility or resistance to damage. Here, we analyzed skeletal neuromuscular junctions (NMJs) in 16 mouse muscles to seek such differences. We find that muscles respond in one of three ways to aging. In some, including most limb and trunk muscles, age-related alterations to NMJs are progressive and extensive during the second postnatal year. NMJs in other muscles, such as extraocular muscles, are strikingly resistant to change. A third set of muscles, including several muscles of facial expression and the external anal sphinter, succumb to aging but not until the third postnatal year. We asked whether susceptible and resistant muscles differed in rostrocaudal or proximodistal position, source of innervation, motor unit size, or fiber type composition. Of these factors, muscle innervation by brainstem motor neurons correlated best with resistance to age-related decline. Finally, we compared synaptic alterations in normally aging muscles to those in a mouse model of amyotrophic lateral sclerosis (ALS). Patterns of resistance and susceptibility were strikingly correlated in the two conditions. Moreover, damage to NMJs in aged muscles correlated with altered expression and distribution of CRMP4a and TDP-43, which are both altered in motor neurons affected by ALS. Together, these results reveal novel structural, regional and molecular parallels between aging and ALS.

## Introduction

Normal aging and age-related neurodegenerative diseases both impair neuronal function. The functional decline observed in aging humans and experimental animals was once thought to reflect, in large part, neuronal death. Over the past decade, however, it has become clear that relatively few neurons die during normal aging [1]; instead, other cellular pathologies predominate of which synaptic dysfunction appears to be a major contributor [1], [2], [3], [4], [5], [6], [7], [8]. In contrast, neuronal death is a prominent feature - in fact the defining feature - of neurodegenerative diseases [9], [10], [11], [12]. Nonetheless, even in these disorders, synaptic dysfunction often precedes and in some cases may even lead to neuronal death. Thus, it is important to explore relationships of neurodegenerative diseases to normal aging, not only because age is their major risk factor but also because they involve synaptic dysfunction.

One striking feature of neurodegenerative disease is that each one selectively affects particular neuronal populations, even when the causal genes are broadly expressed. For example, familial forms of Huntington's disease, Parkinson's disease, Alzheimer's disease and amyotrophic lateral sclerosis (ALS), lead predominantly to death of striatial spiny neurons, dopaminergic substantia nigra neurons, entorhinal cortex and hippocampal neurons, and spinal motor neurons respectively, even though the mutated genes in each condition –i.e. huntingtin, synuclein, APP/tau, and superoxide dismutase 1 (SOD1) - are expressed in most neurons [12], [13]. In some cases, the selectivity of neuronal susceptibility is not only cell-type specific but can differentially affect different classes of the same neuronal type. For example, in familial ALS, motor neurons innervating limb and trunk muscles are severely affected, whereas those innervating extraocular muscles and the anal sphincter muscle are largely spared [14], [15].

Selective effects of aging on synaptic subpopulations have been less well studied.

Comparison of affected and spared synapses within the same region or broad type could aid in the identification of factors that confer resistance to age-related change and, by extension, to neurodegenerative diseases. Here, we have focused on skeletal neuromuscular junctions (NMJs) to undertake such a comparison. Their large size and accessibility facilitates detailed examination of their structural integrity by light microscopy, thereby permitting large numbers of synapses to be sampled. In fact, age-related structural changes in NMJs of limb and trunk muscles have been documented in several mammalian species including mice and humans [7], [16], [17], [18], [19]. We therefore asked whether the rates and patterns of change varied among muscles, and found that they did.

We then asked whether muscles in which NMJs were resistant or susceptible to aging differed systematically in other respects. We considered the rostrocaudal and proximodistal position of the muscle, the myosin heavy chains that its fibers expressed (which define fiber type), the location of the MN pools innervating each muscle, and the size of the motor unit (the number of muscle fibers that each motor neuron innervates). Of these factors, innervation by brainstem motor neurons through cranial nerves correlated best with resistance to age-related synaptic disassembly.

Finally, we compared NMJs in old mice with those in a mouse model of amyotrophic lateral sclerosis (ALS). We report similar patterns of susceptibility and resistance in these two conditions, and extend the structural analysis to document differential effects on levels and distribution of two proteins that have been implicated in the pathogenesis of ALS, CRMP4a and TDP-43 [20], [21]. Our results reveal novel parallels between normal and pathological aging, and provide starting points for seeking factors that could attenuate or minimize synaptic damage in the two conditions.

## Results

### NMJs in extraocular muscles are resistant to age-related structural changes

In a previous study, we documented age-related structural alterations in the NMJs of three hind-limb muscles, the tibialis anterior, gastrocnemius, and gracilis [19]. Alterations were qualitatively and quantitatively similar in all three muscles. To begin the present study, we asked whether NMJs in all muscles age in similar ways. We initially compared NMJs in the extensor digitorum longus (EDL) of the hindlimb with those in extraocular muscles (EOMs). We chose EDL because it showed similar age related changes to the hindlimb muscles previously described [19]. In contrast, EOMs have been reported to resist damage brought about by ALS as well as Duchenne's muscular dystrophy [14], [15], [22].

**Figure 1:**
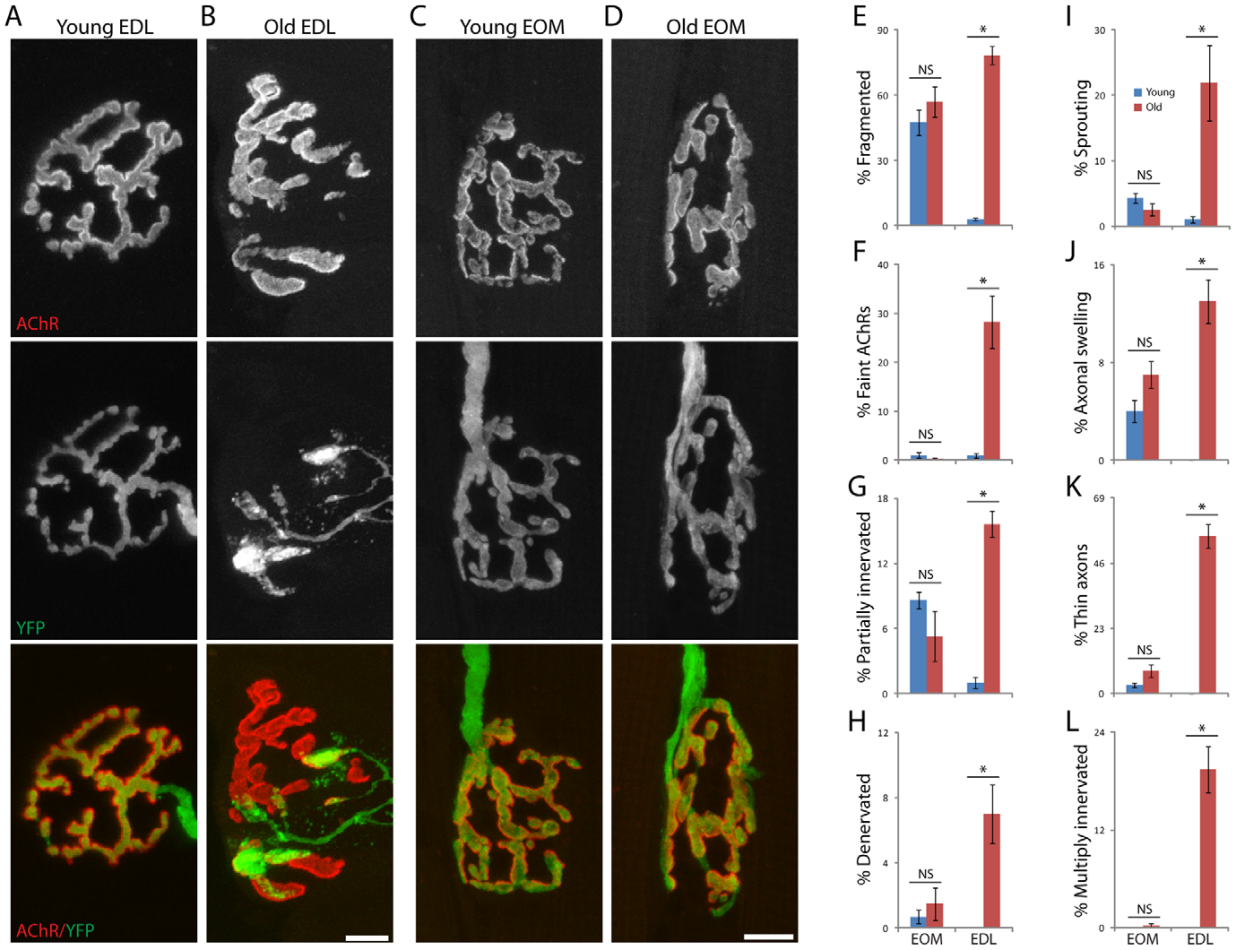
NMJs in young adult and old extensor digitorium longus and extraocular muscles. Muscles from transgenic mice that expressed YFP in axons (green) were stained with BTX to label AChRs in the postsynaptic membrane (red). A) Young adult extensor digitorum longus (EDL). B) Two year-old EDL. C) Young adult extraocular muscle (EOM). D) Old EOM. Age-related alterations are striking in EDL but subtle in EOM. Scale Bar: 10 µm. Eight previously documented age-related alterations [19]were quantified from images such as those shown in Figure 1. E) Fragmentation of the postsynaptic membrane. F) Decreased AChR density. G) Partial denervation. H) Complete denervation. J) Nerve terminal sprouting. I) Preterminal axonal distension. K) Axonal dystrophy. L) Multiple innervation of a single postsynaptic site. Each bar represents mean ± SEM from at least 3 animals, with at least 100 NMJs counted per animal. *p<0.01 by *t*-test. Scale bar = 10 µm.

We cut longitudinal sections of EDL and EOM from young adult (3–6 months old unless otherwise noted) and old (24–28 months old unless otherwise noted) transgenic mice in which motor axons were labeled with YFP [19], [23]. Sections were counterstained with α-bungarotoxin (BTX), which binds specifically to AChRs in the postsynaptic membrane, thereby marking synaptic sites. Nearly 90% of NMJs in the EDL of old mice but only 3% of NMJs in the EDL of young mice (Fig. 1A–B and E–L) exhibited one or more of the following eight features: (1) fragmentation of AChR-rich postsynaptic membrane into small islands; (2) decreased AChR density in some or all of the postsynaptic membrane; (3) retraction of the nerve from the postsynaptic apparatus resulting in partial or (4) complete denervation; (5) sprouts arising from nerve terminals that extended beyond the postsynaptic apparatus; (6) swelling or distension in preterminal axons, within 50 µm from NMJs; (7) thinning of preterminal and/or terminal portions of the axon, termed axonal dystrophy; (8) convergence of 2 or more axons on a single postsynaptic site, leading to multiple innervation. These anatomical changes are similar to those reported previously for tibialis anterior, gastrocnemius and gracilis muscles [19]. NMJs in the EOMs (lateral and medial rectus and inferior and superior oblique), in contrast, were strikingly spared (Fig. 1C–D and E–L). The postsynaptic membrane of most old EOM NMJs was fragmented into small islands, but this topology is also a common feature of young adult EOM NMJs (Figure S1), and is therefore not a sign of aging in this muscle. Values were significantly lower for all 7 other age-related changes in old EOM NMJs than in old EDL (Fig. 1F–L). Thus, NMJs in the EOMs resist age-related structural decline.

### Intermuscular variations in susceptibility to aging

**Figure 2:**
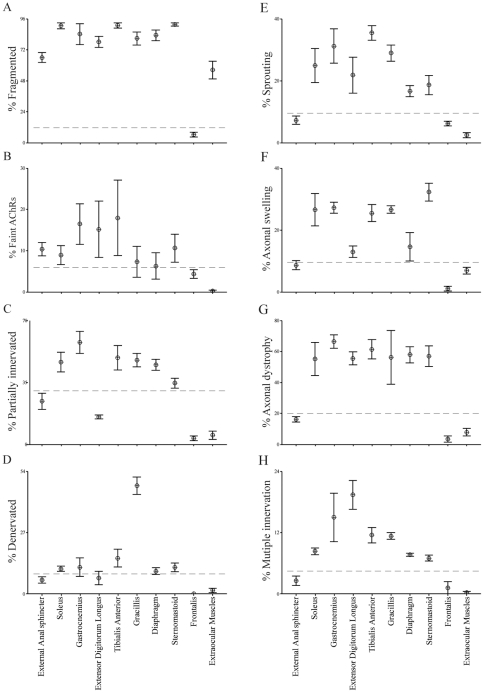
Age-related alterations in neuromuscular junction of 2 year-old mice. Incidence of age-related alterations shown in NMJs from sternomastoid, Gracilis, Soleus Diaphragm, Gastrocnemis, Anal Sphincter, EDL and EOM. A) Fragmentation of the postsynaptic membrane. B) Decreased AChR density. C) Partial denervation. D) Complete denervation. E) Nerve terminal sprouting. F) Preterminal axonal distension. G) Axonal dystrophy. H) Multiple innervation of a single postsynaptic site. Values from Fig. 1 are replotted for comparison. Each point represents mean ±SEM from at least 3 animals, with at least 100 NMJs counted per animal. Dashed lines are drawn to emphasize that values for external anal sphincter, frontalis and EOM are generally lower than those from other muscles.

Are EOM NMJs unique in their resistance to age-related changes? To address this issue, we analyzed NMJs in 16 other muscles of young adult and old mice. For 8 of 16 muscles, we quantified all of the features enumerated above (Figure S2 and Figure 2); for the others, we quantified five of these features: postsynaptic fragmentation, decreased AChR density, partial and complete denervation, and nerve terminal sprouting (Table 1).

**Table 1:** Fraction of NMJs exhibiting one or more age-related alterations.

*Muscle*	*(A)Young*	*(B) Old*	*(C) Young*	*(D) Old*	*(E) Net change, D-C*
Anal sphincter	4	66	0	32	32
Diaphragm	4	83 (4.3)	3	58 (5)	55
Dilator Nari	55 (3.8)	64 (7.6)	35 (1.8)	46 (4)	11
Extensor Digitorum Longus	3	87 (3.8)	2 (0.6)	72 (3.7)	70
Extraocular Muscles	47 (5.8)	57 (6.9)	10 (1.4)	11 (3.1)	1
Frontalis	6 (2)	12 (2)	3 (1)	9 (1)	6
Gastrocnemius	6	94 (2.7)	4	69 (3.2)	65
Gracillis	2	81 (5)	1	61 (13.8)	60
Interscutularis	27 (2.2)	39 (1.2)	22 (1.5)	31 (2)	9
Levator Auris Longus	19 (2.7)	35 (4.5)	13 (2.3)	21 (1.7)	8
Levator labii superioris	29 (5.3)	46 (1.7)	16 (2.9)	37 (2.5)	20
Omohyoid	15 (2.4)	76 (5.3)	11 (0.6)	52 (2)	42
Parotidoauricularis	24 (5)	31 (4)	15 (2)	23 (2)	8
Soleus	10	91 (3.8)	2	55 (10.7)	53
Sternomastoid	2 (0.4)	92 (2)	2 (0.3)	57 (6.7)	55
Sternomyoid	11 (2.1)	89 (3)	8 (3)	75 (3.8)	67
Tibialis Anterior	2	94 (2.6)	2	65 (5.8)	63
Triangularis	7 (.8)	87 (2.3)	4 (1.3)	68 (3)	64

Columns 2 and 3 show the percentage of NMJs in young adult and old muscles that exhibit one or more of five structural features that characterize old NMJs in limb muscles: decreased AChR density, partial denervation, complete denervation, nerve terminal sprouting, fragmentation of the postsynaptic membrane. Column 4 and 5 show values for only the first four of these features, taking account of the fact that the postsynaptic membrane is fragmented in young adult NMJs in some muscles. Column 6 shows difference between values in columns 4 and 5. Values represent average (and SD) from at least 3 animals, with at least 100 NMJs counted per animal.

Muscles varied greatly in the incidence of age-related changes in their NMJs. In some muscles, such as the soleus of the lower hindlimb, the gracilis of the upper hindlimb, the diaphragm in the trunk and the sternomastoid in the neck, alterations were similar in frequency to those documented above for EDL (Fig. 2A–H). At the other extreme, muscles such as the external anal sphincter (EAS) and the frontalis were largely spared (Fig. 2A–H).

**Figure 3:**
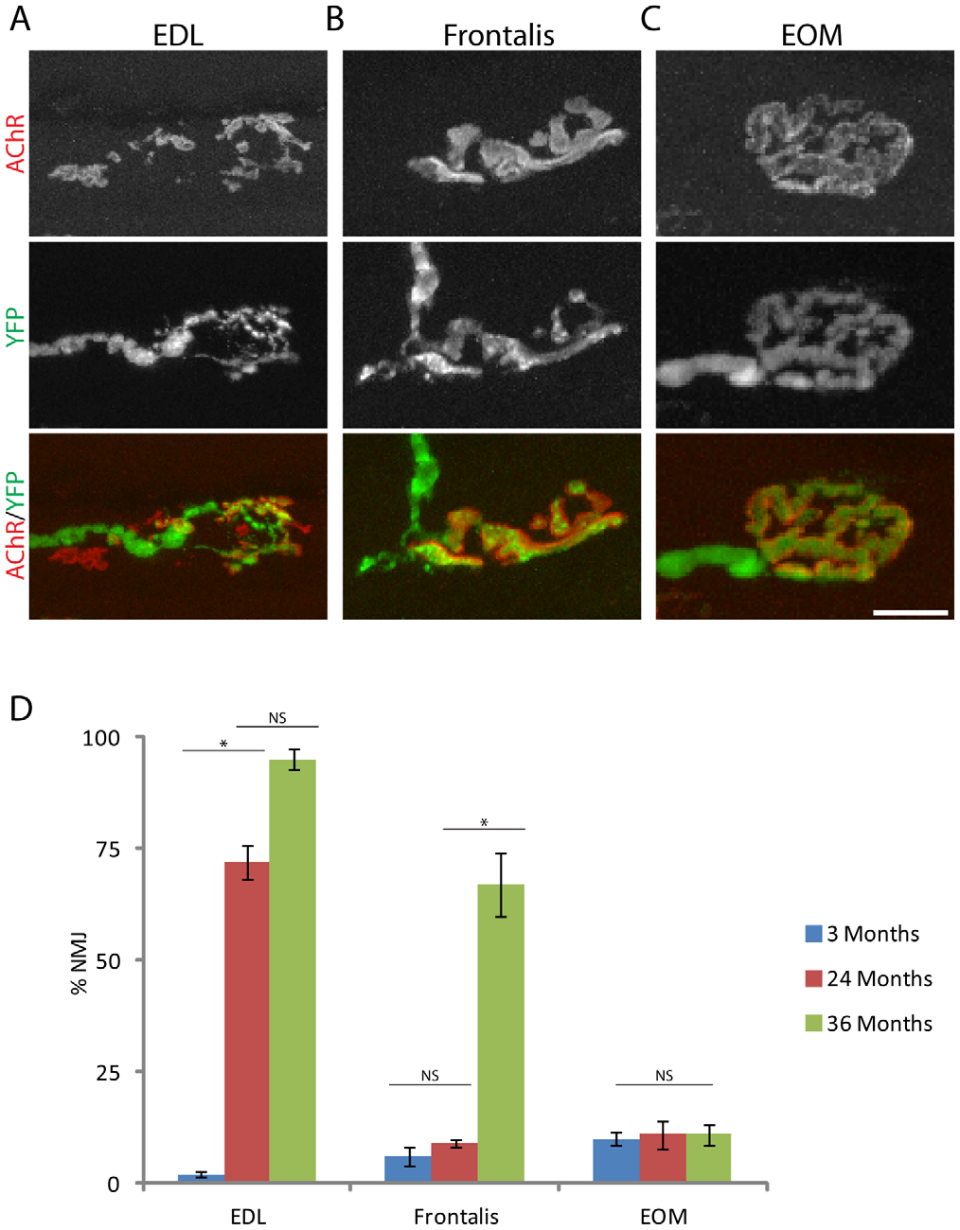
Structural alterations in NMJs during the third year. NMJs from EDL (A), frontalis (B) and EOM (C) of 3 year-old mice. D) Incidence of age-related alterations in NMJs of 3 month-, 2 year-, and 3 year-old mice. Each bar represents mean ± SEM from at least 3 animals, with at least 100 NMJs counted per animal. *p<0.025 by *t*-test. Scale bar = 10 µm.

These differences might reflect absolute differences in susceptibility among muscles or differences in the rates at which defects accumulate. To distinguish between these two possibilities, we examined NMJs in 3 year-old mice. The incidence of affected NMJs changed little in the third year for the EDL, although qualitatively, NMJs appeared more seriously affected in 3 year-old mice than in 2 year-old mice (Fig. 3A, D). Likewise, NMJs in EOMs of 3 year-old mice resembled those in 2 year-old mice, with few structural signs of aging at either time (Fig. 3C, D). In contrast, the structure of NMJs in the frontalis changed dramatically during the third year: they were nearly as youthful in appearance in 2 year-old mice as those in EOMs, but the incidence of structural alterations increased ∼5-fold during the third year, approaching that in the EDL (Fig. 3B, D). Based on these results, we analyzed additional muscles at three years of age including the EAS and other muscles of facial expression (interscutularis, levator auris longus [LAL], and levator labii superioris [LLS]) and an accessory EOM which elevates the eyelid (levator palpebrae surperioris [LPS]). NMJs in the LPS, like those in other EOMs, remained spared at three years of age, whereas those in the EAS, interscutularis, LAL, and LLA accumulated defects over the third year (data not shown). Thus, there may be three groups of muscles with different susceptibility to aging: those highly affected by two years of age (such as EDL), those that succumb during the third year and, therefore, have a delayed response to aging (facial and sphincter muscles) and some that are largely spared (EOMs and LPS).

### Rostrocaudal position and segmental innervation

What could account for intermuscular differences in the susceptibility of NMJs to the age-related decline? Muscles differ in many ways that might be correlated with susceptibility, including their position along the rostrocaudal axis, whether they are innervated by spinal or cranial motor neurons, their fiber type composition, the size of their motor units and the length of the axons supplying them. Indeed, the highly susceptible EDL and the spared EOM differ in all of these respects [24], [25], [26], [27], [28]. First, we used the data in Table 1 to assess the relationship between the segmental origin of the nerves that innervate each muscle and the incidence of age-related alterations in their NMJs. We used segmental innervation as a metric because it is a correlate of the muscle's somite origin, which is unknown in many cases, and of body position, which is difficult to specify in a single dimension.

**Figure 4:**
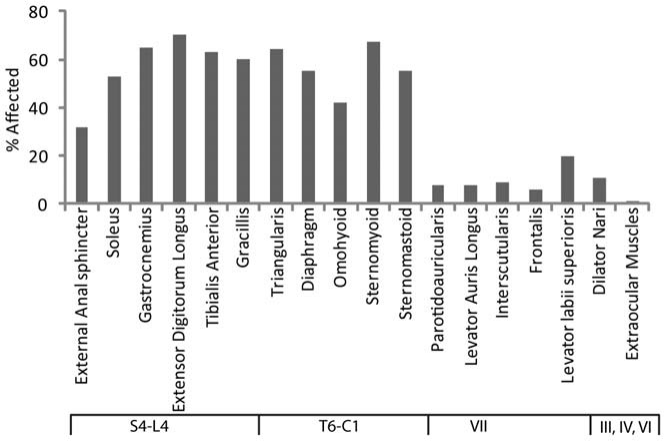
Relationship of rostrocaudal position and segmental innervation to the incidence of age-related alterations in NMJs. Muscles listed in Table 1 are arranged by the rostrocaudal position of the motor pool that innervates them. The ordinate is the percentage of NMJs with age-associated defects from column 6 of Table 1.

In general, NMJs in rostral muscles showed less age related changes than those in caudal muscles (Fig. 4). However, hindlimb and neck muscles are similarly affected by aging even though they are innervated by motor neurons located in caudal and cervical portions of the spinal cord, respectively. Moreover, the EAS appeared spared at two years of age despite being quite caudal. Thus rostrocaudal position per se is not directly related to the incidence of age-related changes. On the other hand, there is a striking difference between muscles innervated by brainstem motor neurons (through cranial nerves) and those innervated by spinal motor neurons: the incidence of age-related changes in NMJs was higher in all muscles innervated through spinal nerves than in muscles innervated by cranial nerves III, IV, VI, and VII. Thus, NMJs formed by brainstem motor neurons are generally refractory to age-related defects.

### Fiber type composition

In most skeletal muscles, fibers are divided into 4 categories based primarily on the particular myosin heavy chain (MyHC) isoform they express - type I, IIA, IIX or IIB, ranging from slowest to fastest [26]. Individual muscles contain stereotyped proportions of two or more fiber types. Several studies have shown that fast fibers are more susceptible than slow fibers to atrophy and denervation within aging muscles [29]. It therefore seemed plausible that the degree of severity to age related alterations in some muscles is largely dependent on their fiber type composition. Consistent with this idea, many fibers in the highly resistant EOM express an unusual myosin heavy chain, *myh13* [30], [31].

**Figure 5:**
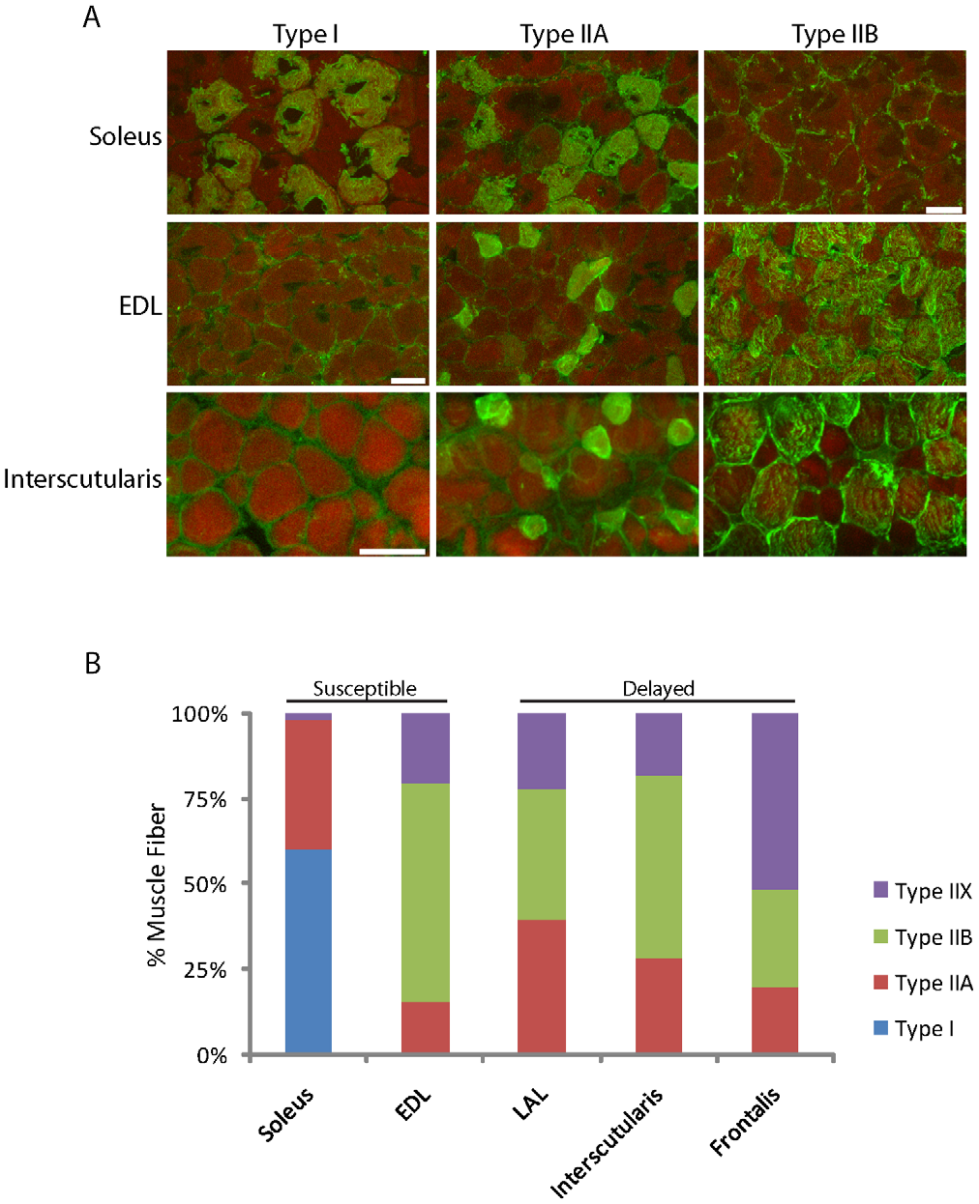
Fiber type composition of muscles with NMJs that vary in severity of age-related changes. A) Cross-sections of soleus, EDL, and interscutularis muscles from young adult mice stained with antibodies specific for myosin Type I, IIA or IIB. Scale Bar: 20 µm. B) Fiber type composition of muscles, determined from micrographs such as those in A. NMJs in both soleus and EDL suffer severe age-related changes but differ in fiber type composition. In contrast, NMJs in frontalis, levator auris longus and intercutularis are largely spared from age-related changes, even though their fiber type composition of these muscles is similar to that of the EDL.

We used a panel of isoform-specific antibodies to myosin heavy chains to analyze the fiber type composition in a set of young adult muscles that vary in susceptibility to aging. The frontalis, LAL and interscutularis muscles, which exhibit age-related changes only during the third year have a similar fiber type composition to the EDL, which is highly susceptible to age related changes (Figure 5). In contrast, the soleus, with nearly all slower fibers (>90% Type I and IIA) and the EDL, with predominantly fast fibers (>80% Type IIX and IIB) are similarly susceptible (Figure 5 and Table 1). This data suggest that the overall fiber type composition of muscles does not impart resistance.

**Figure 6:**
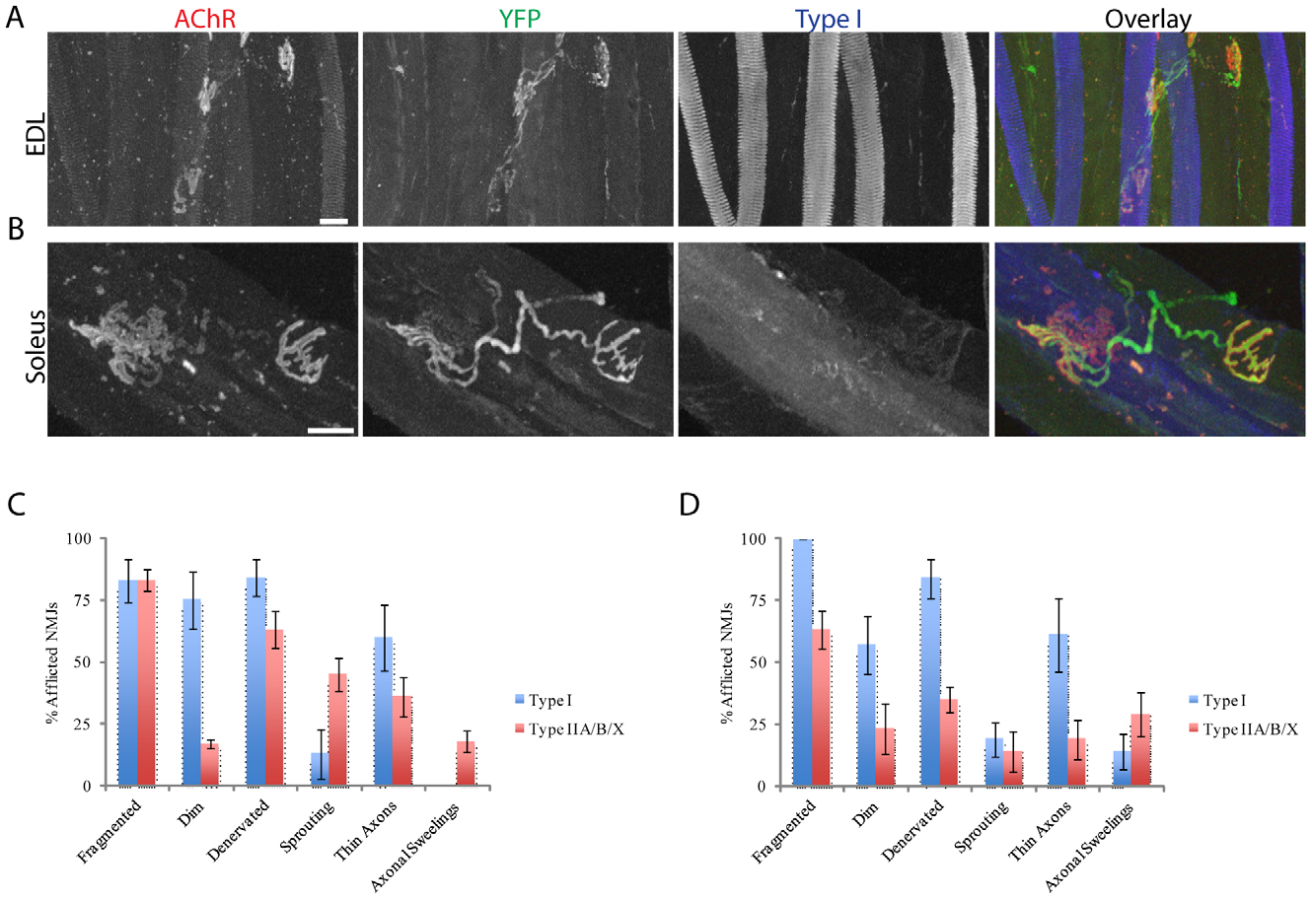
NMJ morphology in Type I and Type II muscle fibers of old mice. A, B) EDL and Soleus muscles from 2 year-old mice were stained for Type 1 muscle fibers. Type 1 muscle fibers are found in old (A) but not young EDL muscles (see Fig. 5). C, D) NMJs in Type I and Type II fibers exhibit age-related changes in EDL (C) and soleus (D). Each bar represents mean ± SEM. Scale bar = 20 µm.

The fiber type composition of fast type muscles is known to change with aging, with fast (Type II) converting to slow (Type I) muscle fibers [26]. The switch to a more oxidative type is believed to allow muscle fibers to resist aging. We therefore asked if a high rate of fiber type switching may account for the special resistance of NMJs in muscles such as the frontalis and interscutularis muscles, which are composed of type IIA, IIB and IIX muscle fibers in young adult animals. We stained 2-year old frontalis, interscutularis and EDL muscles for type I muscle fibers. In contrast to young muscle, aged EDL muscle contains a significant number of Type I fibers (Fig. 6A, Fig. 5A and B). However, fiber type composition did not change significantly in aged frontalis or interscutularis muscles (data not shown). Thus, fiber type switching does not appear to account for the resistance of NMJs in facial muscles, at least during the first two years.

We also asked whether the severity of age-related changes in NMJs within single muscles might be related to fiber type. Unfortunately, we could label only Type I fibers in the fixed whole mounts that are required to assess NMJ architecture. In the soleus >95% of fibers, however, are either Type I or IIA fibers, so nearly all unlabeled fibers were Type IIA fibers. Using this approach we were surprised to discover that Type I fibers exhibited a higher incidence of age-related structural defects in their NMJs than Type IIA fibers in the soleus muscle (Fig. 6B and D). We also used the EDL to compare the incidence of age-related changes between fibers that converted to Type I with other fiber types. The rate of age-related changes in NMJs was similar in Type I (labeled) and Type II fibers (unlabeled) of old EDL (Fig. 6A, C). Hence, particular fiber types do not appear to be endowed with unique abilities to protect their NMJs from aging.

### Motor unit size

**Figure 7:**
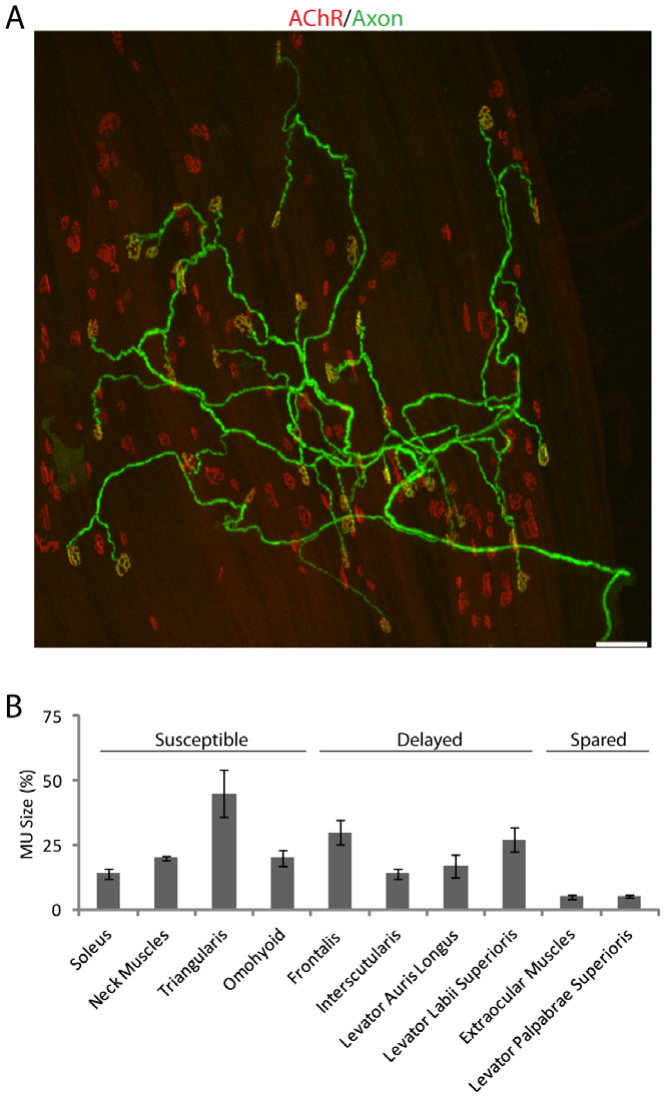
Motor unit size in muscles with NMJs that vary in severity of age-related changes. A) Frontalis muscle of a young adult mouse in a simple motor axon was YFP-positive (green). The muscle was also labeled with BTX (red) to mark all postsynaptic sites. Scale bar: 50 µm. B) Motor unit size determined from muscles such as that in A. Bars indicate mean (±SD) of number of motor units in parentheses. Data on neck muscles (sternomastoid, clavotrapezius, and cleidomastoid) are replotted from Schaefer et al., (2005).

If motor neurons have limited capacity to maintain nerve terminals, those that innervate many muscle fibers may be at greater risk of losing terminals as their capacity declines with age than those that innervate few fibers. If true, muscles with small motor units might be more resistant to age-related decline than those with large motor units. To test this idea, we used transgenic mice that express YFP in only a small subset of motor axons (*thy1-YFP line H*) [23], [32] to reconstruct entire axonal arbors of individual motor axons in muscles that vary in susceptibility to age-related changes. Figure 7A shows one example and Figure 7B summarizes data from all young adult muscles analyzed here and in previous studies from our laboratory [33], [34]. Consistent with previous reports [27], EOM motor units are small, averaging 5 fibers. Likewise, the LPS, also spared, has motor units of approximately 5 fibers [35]. Conversely, limb muscles, triangularis and omohyoid, all of which are susceptible, have motor units averaging 14 to 45 muscle fibers (Fig. 7B). However, motor units in the frontalis, interscutularis, LLS and LAL muscles, which are largely spared at 2 years of age, are similar in size to those found in omohyoid and limb muscles. Thus, muscles with the smallest motor units are the most resistant to aging, but small motor units are not required to endow NMJs with resistance to aging. Specifically, motor unit size cannot account for the difference between muscles that are most susceptible to age-related changes and those that exhibit such changes only during the third year.

**Figure 8:**
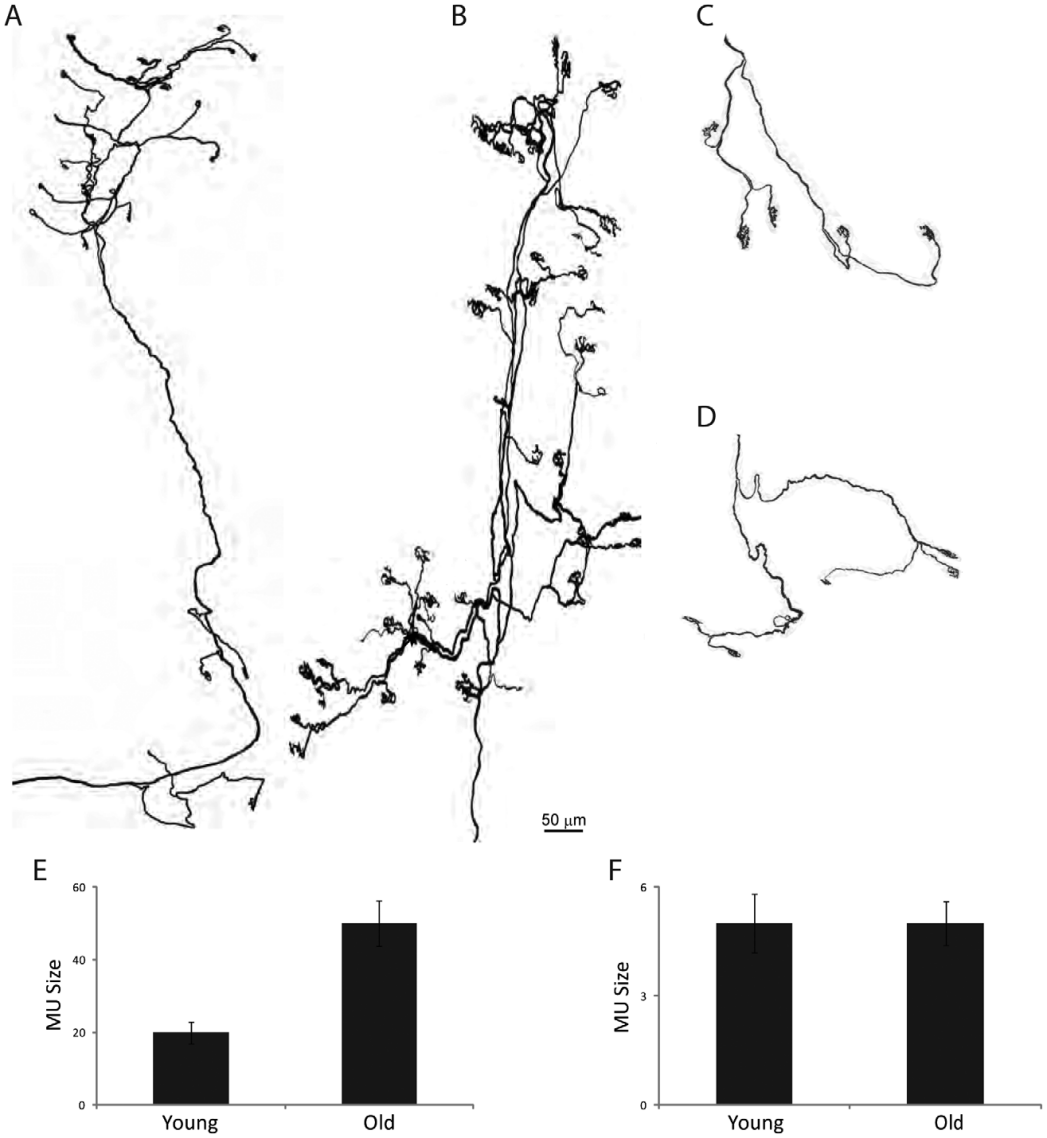
Age-related changes in motor unit size. Tracings from muscles such as the one shown in Figure 7A. A–D) Motor units from the omohyoid (A, B) and extraocular muscles (C, D) of young adult (A, C) and old (B, D) mice. E, F) Motor unit size in young adult and old omohyoid (E) and extraocular (F) muscles. Each bar represents mean ± SEM from at least 4 motor units per muscle and age. Scale bar = 50 µm.

We also examined motor units in old muscles. Motor units have been reported to expand during normal aging likely due to sprouting of surviving motor units to innervate synaptic sites left denervated by death of other motor neurons [36]. Consistent with this view, motor units in the omohyoid were on average 2.4 fold larger in old than in young adult muscles (Fig. 8A–C). In contrast, the size of motor units in EOMs, which are resistant, did not increase significantly with age (Fig. 8D–F). Similarly, motor unit size changed little in several facial muscles that are largely spared, including the frontalis, interscutularis, LAL and LLS muscle (not shown).

### Proximodistal position

**Figure 9:**
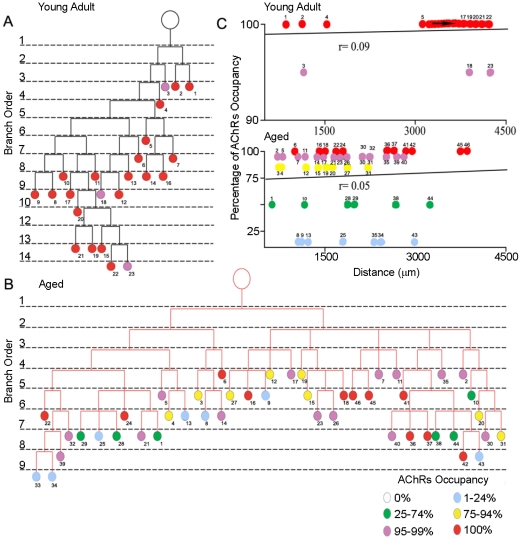
Branch order analysis of motor axons in young adult and aged animals. A, B) Branching trees from motor units in young adult (A) and old (B) omohyoid muscles. Color of circles represents the degree of AChR occupancy by nerve terminals (color code, bottom right corner). C) No correlation between AChR occupancy and distance of the nerve terminal from the first branch point of the axonal arbor in either the young adult (r = 0.09) or the aged animal (r = 0.05).

The ability of neurons to transport material to their terminals decreases with age [37], [38]. Muscle fibers innervated by long motor axons might be more affected by this decrease than those with short motor axons. Two observations suggested, however, that this relationship is not a robust one. First, we compared neck muscles, such as the omohyoid, to lower limb muscles such as the tibialis anterior and soleus. Neck muscles are innervated by shorter axons than lower limb muscles, but NMJs in neck and limb muscles are similarly affected by aging (Table 1). Second, we compared NMJs in proximal and distal portions of single motor units in old muscles. NMJs within motor units varied substantially in structure with some appearing normal and others being almost completely denervated (Figure S3). However, severely affected NMJs were distributed throughout the motor unit, and were not especially prevalent in proximal or distal axonal branches (Figure 9).

### Correlated susceptibility of NMJs to age and ALS

Structural alterations of NMJs have been documented in patients with ALS and in mouse models of ALS that overexpress mutant forms of superoxide dismutase-1 (SOD), known to cause dominant familial ALS in humans [39], [40]. Mice overexpressing the pathogenic human SOD-G93A show motor symptoms at around 14–16 weeks of age (symptomatic stage) and die at 19 weeks of age (end-stage). Synaptic defects in symptomatic SOD-G93A mice resemble those that afflict NMJs in old mice in several respects [34]. Moreover, two muscles that are spared in old mice, the EOMs and anal sphincter, are resistant in humans with ALS [15], [41].

**Figure 10:**
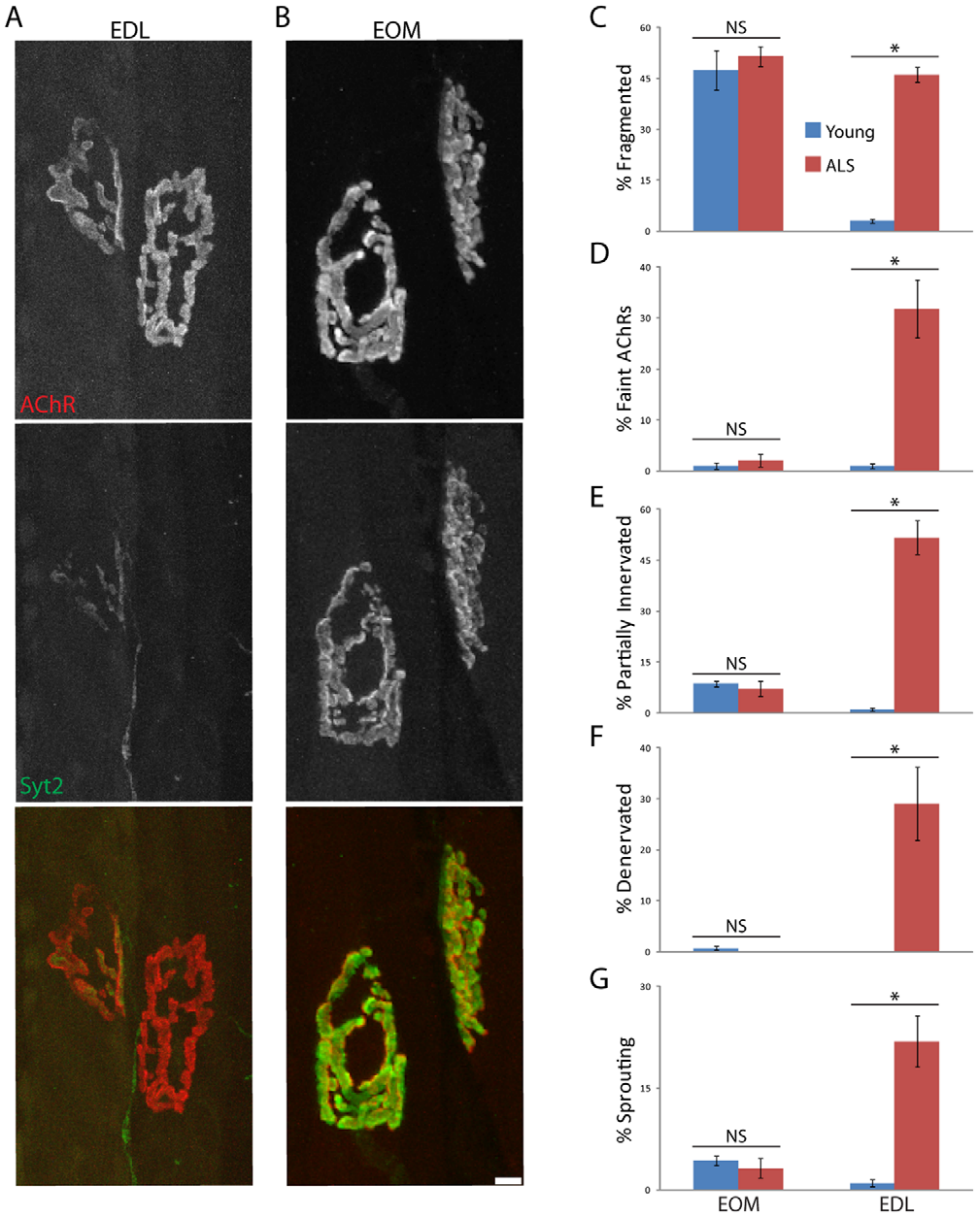
Neuromuscular junctions in a mouse model of ALS. A, B) Longitudinal sections from EDL (A) and EOM (B) of SOD-G93A mice transgenic mice. Axons and nerve terminals were stained with antibodies against neurofilament and synaptotagmin-2, (green) and postsynaptic sites were stained with BTX (red). Compare with young adult controls in Figure 1 and Figure S1. Alterations are striking in EDL but subtle in EOM. C) Alter ations quantified from images such as those shown in A–B. Each bar represents mean ± SEM from at least 3 animals, with at least 100 NMJs counted per animal. *p<0.02 by *t*-test. Scale bar = 10 µm.

Based on these parallels, we analyzed intermuscular variations in the NMJs of SOD-G93A mice. NMJs in EDL of SOD-G93A mice exhibited the defects described above for NMJs in EDL of old mice: postsynaptic fragmentation, decreased AChR density, partial and complete denervation, terminal sprouting, multiple innervation, and axonal distension and dystrophy (Fig. 10A, C–G). In contrast, NMJs in EOMs of SOD-G93A mice were largely spared (Fig. 10B–G).

**Figure 11:**
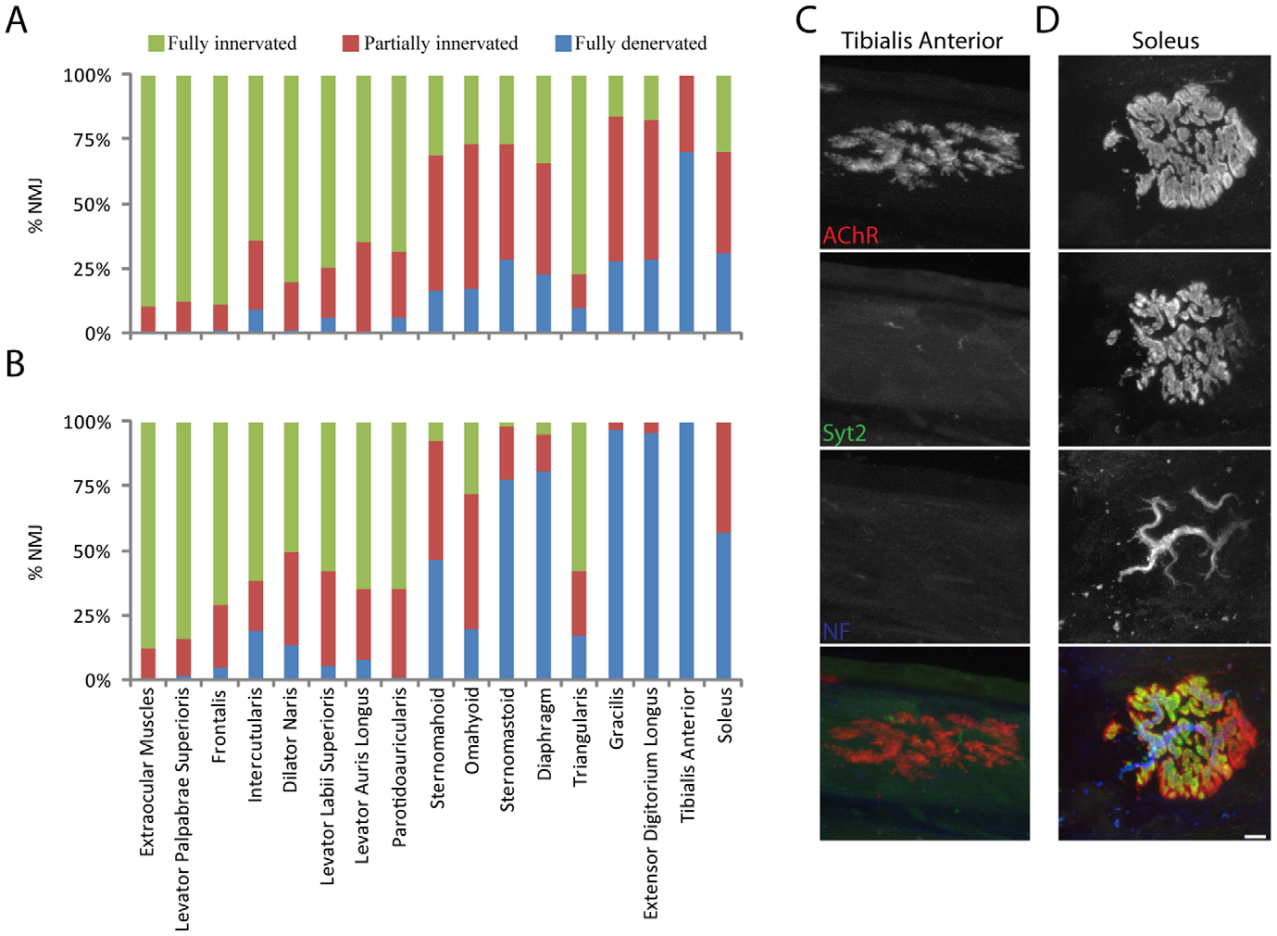
Extent of denervation in muscles from SOD-G93A mice. A, B) NMJs in muscles from symptomatic (A) or end-stage (B) SOD-G93A mice listed were scored as fully innervated, partially denervated or fully denervated. Values are shown for 14–16 weeks old symptomatic (A) and 19 weeks old end-stage mice (B). C) NMJs from soleus and tibialis anterior muscles of end stage mice, stained as in Fig. 9. Scale bar = 10 µm. Some NMJs remain partially innervated in soleus, but most NMJs are fully denervated with dispersed postsynaptic structures in tibialis anterior.

We also determined the fractions of synaptic sites that were partially or completely denervated in symptomatic SOD-G93A transgenic mice for each of the muscles previously studied in old mice. Results are summarized in Figure 11A. At the symptomatic stage, most NMJs in all hind limb muscles studied were partially or fully denervated. Most trunk muscles (with the exception of the triangularis sterni) were also denervated although to a lesser extent than hind limb muscles. In contrast, most NMJs in facial muscles remained innervated and thus were refractory to the effects of ALS. Similarly, most NMJs in the anal sphincter muscle were innervated in SOD-G93A symptomatic animals (data not shown). NMJs in EOMs, LPS and frontalis muscles were almost completely spared.

**Figure 12:**
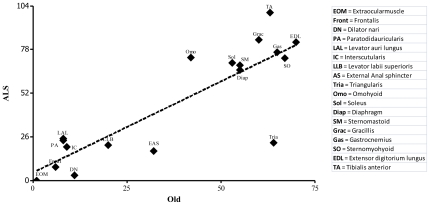
Relationship between the incidence of NMJ alterations in old mice and a mouse model of ALS. Values are from Table 1 and Figure 10. NMJs in all muscles but the triangularis are similarly afflicted or spared by aging and ALS.

We then examined NMJs in mice at the end stage of ALS to determine whether spared muscles eventually succumb (Fig. 11B). Most limb and trunk muscles were almost completely denervated at this stage. Two exceptions were the soleus and triangularis, in which ∼40% and 85% of NMJs, respectively, were still partially or fully innervated. Likewise, NMJs in facial muscles were more affected in endstage than symptomatic ALS. In contrast, the NMJs of the EOMs and LPS muscle remained resistant to disease progression. These results demonstrate that most muscles are similarly affected or spared by aging and ALS (Figure 12).

### Expression of CRMP4a and TDP-43 in aging and ALS

The parallels between aged and ALS-afflicted NMJs raised the possibility that similar molecular alterations occur in motor neurons in both conditions. To test this idea, we focused on two proteins recently found to be altered in spinal motor neurons of SOD-G93A mice, collapsin response mediator protein 4a (CRMP4a) and TAR DNA-binding protein 43 (TDP-43) [20], [21], [42], [43], [44]. In spinal motor neurons of young adult control mice, CRMP4a levels are low and TDP-43 is largely nuclear, whereas in spinal motor neurons of symptomatic SOD-G93A mice, CRMP4a is dramatically increased and TDP-43 forms cytoplasmic aggregates [20], [43], [44]. We assessed the levels and distribution of CRMP4a and TDP-43 in spinal and cranial motor neurons of young adult, old and SOD-G93A mice.

**Figure 13:**
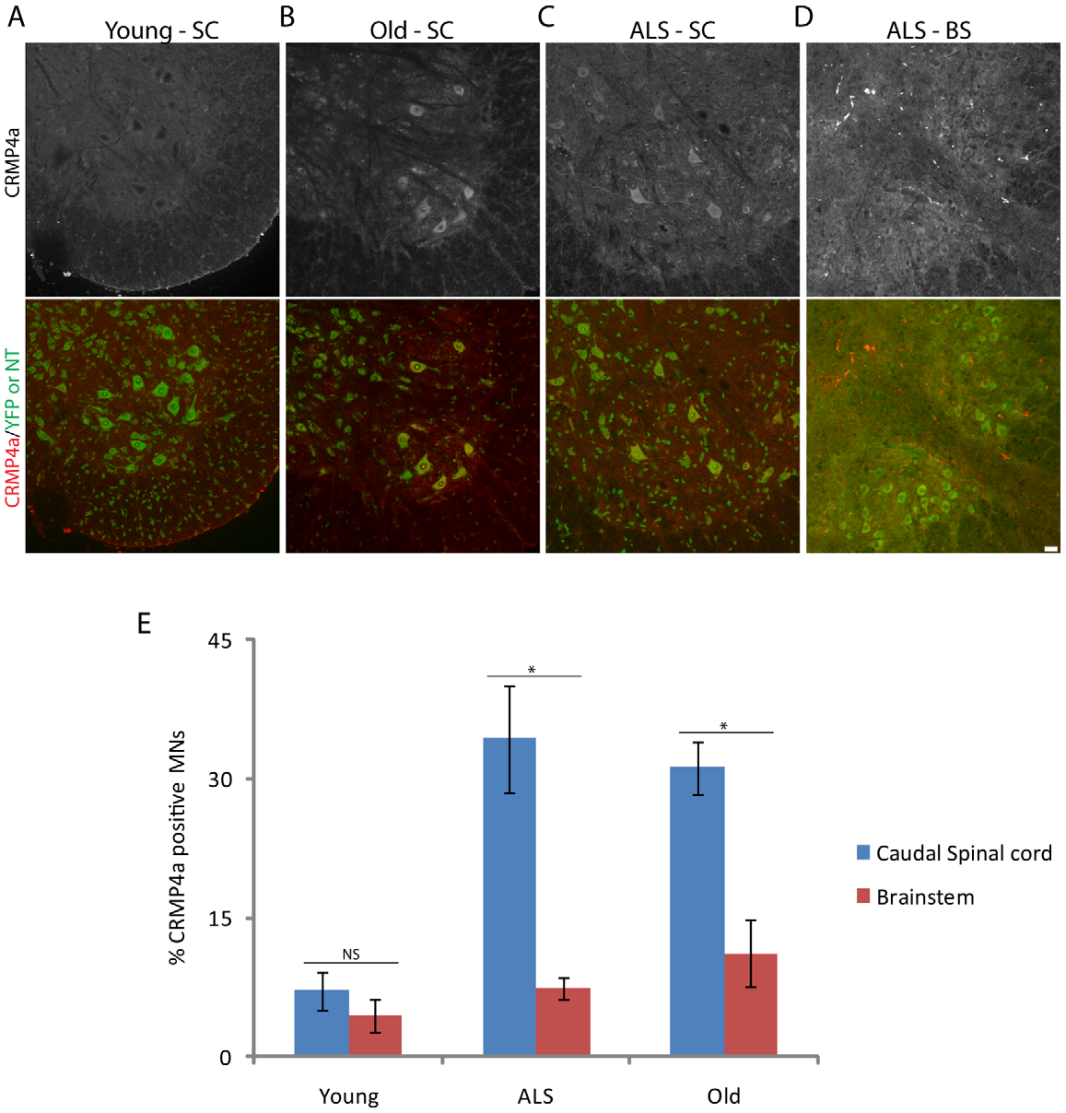
Regulation of CRMP4a in aging and ALS. A–D) CRMP4a immunoreactivity of motor neurons in caudal spinal cord (SC) from young adult wild type (A), old wild-type (B) and symptomatic SOD-G93A (C) mice and brainstem (BS) of a symptomatic SOD-G93A mouse (D). Somata are labeled with Neurotrace (A, B, D) or YFP (C). E) Percent of motor neurons immunoreactive for CRMP4a in spinal cord and brainstem of young adult, old, and symptomatic SOD-G93A mice. Each bar represents mean ± SEM. *p<0.015 by *t*-test. Scale bar = 20 µm.

As reported previously [20], CRMP4a is nearly undetectable in motor neurons in lumbar segments of the spinal cord of control mice, but readily detectable in about half of the lumbar motor neurons of symptomatic SOD-G93A mice (Fig. 13A, C, E). Similarly, the fraction of lumbar motor neurons containing CRMP4a increased ∼5-fold in old mice (Fig. 12B, E). Similar results were obtained for motor neurons in cervical spinal cord (data not shown). In contrast, few cranial motor neurons were CRMP4a-positive in young adult wild-type (3 months), old wild-type (2 years) or symptomatic SOD-G93A (19 weeks) mice (Fig. 13D, E). Thus, differences between spinal and cranial motor neurons and similarities between normal aging and ALS extend to CRMP4a expression.

**Figure 14:**
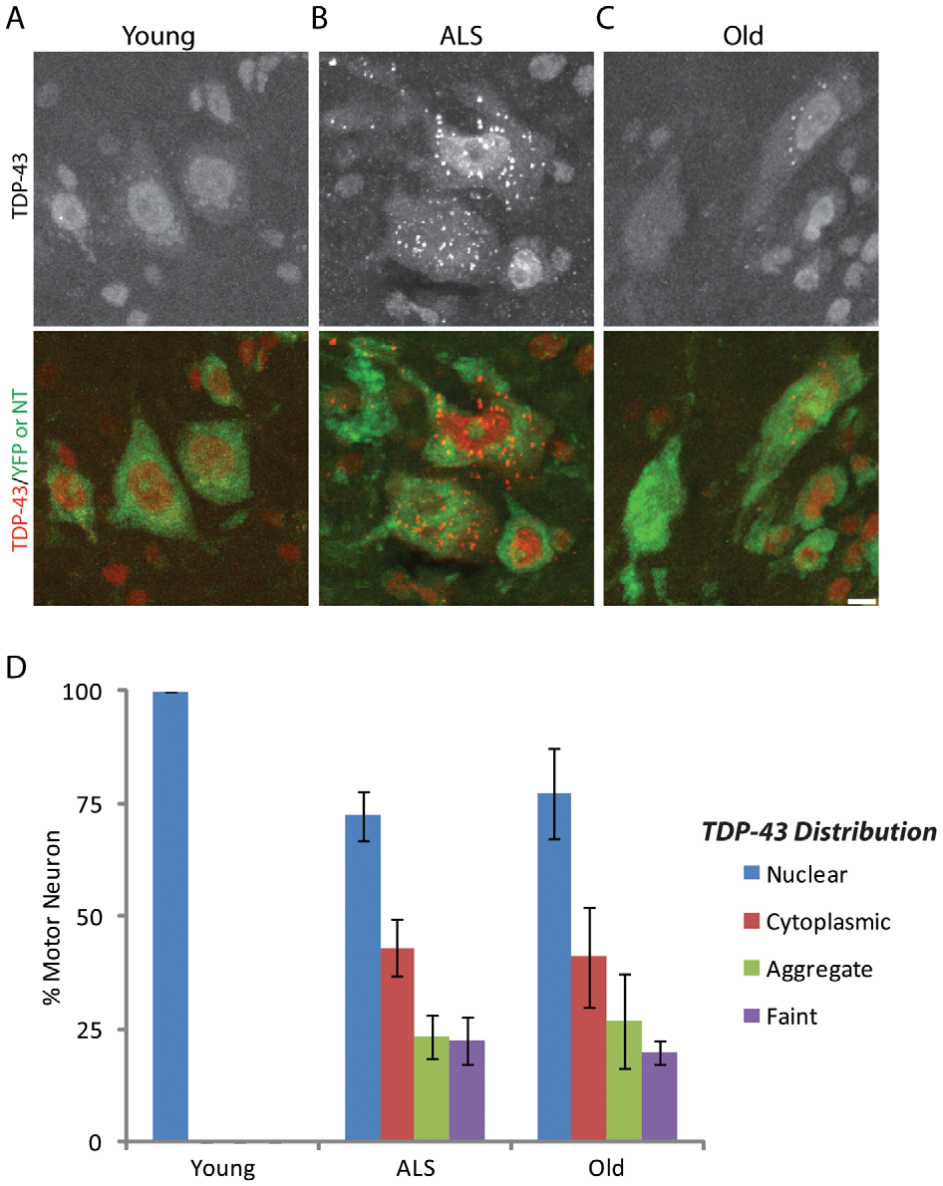
Level and localization of TDP-43 in aging and ALS. A–D) TDP-43 immunoreactivity of motor neurons in caudal spinal cord from young adult wild type (A), old wild-type (B) and symptomatic SOD-G93A (C) mice. Somata are labeled with Neurotrace (A, B) or YFP (C). D) Percent of motor neurons with strong cytoplasmic immunoreactivity, cytoplasmic aggregates, or barely detectable levels of TDP-43 in spinal cord of young adult, old, and symptomatic SOD-G93A mice. Each bar represents mean ± SEM. Scale bar = 20 µm.

Localization of TDP-43 within motor neurons was also differentially affected in cranial and spinal motor neurons by aging and ALS. As reported previously [44], TDP-43 formed cytoplasmic aggregates in the cytoplasm of spinal (lumbar and cervical) motor neurons of symptomatic SOD-G93A mice (Fig. 14B, D). Similar cytoplasmic aggregates of TDP-43 were abundant in spinal motor neurons of old mice (Fig. 14C, D). In addition, whereas levels of TDP-43 were high in all spinal motor neurons of young adult wild type mice (3 months), some motor neurons in SOD-G93A and old mice had barely detectably levels of TDP-43 (Fig. 14D). In contrast, the distribution of TDP-43 in cranial motor neurons from 2-year old and SOD-G93A mice was indistinguishable from that in adults (not shown).

## Discussion

This paper reports two sets of comparisons among NMJs in mouse muscles. First, we compared NMJs within and among 16 muscles of old mice. These comparisons established large intra- and inter-muscular differences in the susceptibility of NMJs to age-related structural alterations. By considering these differences in light of structural and functional features of the muscles and their innervation, we drew conclusions about factors that are likely and unlikely to contribute to differential susceptibility to age-related change. Second, we asked whether the inter-muscular differences in old mice were similar to those in a mouse model of the neurodegenerative disease, ALS. This comparison revealed striking similarities between patterns of susceptibility in these two conditions, strengthening the parallels between normal and pathological aging.

### Differences among muscles in old mice

In a previous study, we found that few NMJs in hind-limb muscles of young adult mice but a majority of NMJs in hind-limb muscles of 2 year-old mice exhibit one or more of eight structural features: fragmentation of the postsynaptic apparatus, reduced levels of AChRs, partial denervation, complete denervation, sprouting of terminal axons beyond the postsynaptic apparatus, swelling of the preterminal axon near or at the NMJ, thinning of the axon within the NMJ, and multiple innervation of a single postsynaptic site [19]. Here, we determined the frequency of these age-related features in a larger set of muscles from both 2- and 3-year old mice. Frequency varied over a wide range, but muscles can be generally grouped into three categories. First, NMJs in all hind-limb, trunk and neck muscles examined are highly susceptible to age-related decline. Although some differences among muscles in this category are statistically significant, they are relatively small. Second, NMJs in extraocular and levator palpabrae muscles (EOMs and LPS) are remarkably resistant to the deleterious effects of old age. Even in 3 year old mice -at which time a majority of animals in the cohort had already succumbed- NMJs in these muscles retained their youthful appearance. Third, NMJs in a set of head muscles innervated by cranial nerves, as well as the external anal sphincter, appeared to be resistant to age-related decline at 2 year of age but succumbed over the following year. Thus, these muscles exhibit a delayed response to aging.

### Similarities between aging and ALS

The structural defects in hind limb NMJs that accumulate during normal aging resemble those that occur in ALS and in mouse models of ALS [19], [34], [45]: in both cases, the postsynaptic membrane becomes fragmented, partially or completely denervated, axons sprout beyond the postsynaptic apparatus, some AChR clusters become faint, and some synaptic sites become multiply innervated. Moreover, NMJs in EOMs are spared in old mice, in humans with ALS and, as shown here, in a mouse model of ALS. These parallels led us to ask whether NMJs in other muscles were similarly susceptible or refractory to disassembly caused by aging and ALS.

The EOM and LPS, as expected, are completely spared in ALS (Fig. 1) [14], [15]. Even at endstage of the disease, NMJs in the EOM and LPS are fully innervated. The external anal sphincter and facial muscles are also partially spared in ALS as they are in old mice. In symptomatic animals, these muscles show little sign of denervation during the initial phase of the disease. As the disease progresses, however, many of their NMJs become denervated. Hence, many muscles are similarly affected by ALS and aging. Interestingly, a few muscles are more susceptible to age than to ALS. Most strikingly, the triangularis muscle remains innervated in ALS even though it is substantially affected in old mice. The soleus muscle has been reported [46] to resist ALS, but we find that it is initially as affected as other hind limb muscles. As the disease progress, however, denervation of the soleus NMJs significantly slows compared to other limb muscles. Hence, the triangularis and soleus appear capable of recruiting mechanisms that act to prevent or retard destruction of NMJs.

### Possible determinants of differential susceptibility to aging and ALS

Why are some NMJs resistant while others are highly sensitive to the deleterious effects of aging and ALS? Among many possible cellular determinants, several seemed reasonable candidates to test. (1) Previous studies have shown that the muscle fiber type composition changes with aging [31], suggesting that the extent of the change could affect susceptibility. Likewise, slow muscles are reported to be relatively spared in ALS [47]. (2) In that motor neurons have restricted ability to supply their terminal branches, one might imagine that small motor units would be better nurtured and thus more refractory to resist aging and ALS. (3) Because the rate of axonal transport declines with age and during the progression of ALS [9], [37], [38], NMJs far from motor neuronal somata might lose sustenance before those close to their neuronal somata. Our experiments addressed each of these possibilities but our results supported none of them. Likewise, although we did not directly assess whether patterns of muscle activity confer resistance or susceptibility to insults indirect evidence suggests that they do not. EOMs are unique in that they are able to fire at exceptionally high rates (∼600 Hz) but are also resistant to fatigue [48]. However, the other muscles spared by aging and ALS do not share this specialization: many fibers in the external anal sphincter and LPS are tonically active [28], [49] and muscles of facial expression exhibit phasic activity similar to those of somite-derived muscles.

Of all the possibilities we considered, our data favors only one. Most muscles that resist age or ALS related decline are innervated by motor neurons that reside in the brainstem and extend axons through cranial nerves. This is especially true for oculomotor, abducens and trochlear motor neurons that innervate the EOMs and the LPS muscle, which are completely spared by aging and ALS. Most facial nuclei motor neurons also resist aging and ALS, but the partial resistance of facial muscles suggests that some motor neurons may be susceptible. Interestingly a recent study showed that NMJs in caudal and rostral LAL muscle are differentially susceptible to denervation in a mouse model of spinal muscular atrophy [50]. Our studies, however, combined data from all regions of the sampled muscles, so we cannot directly compare our results to those in Ref. 50.

Of muscles innervated by cranial nerves, one apparent exception is the sternomastoid which is severely affected by aging and ALS, but innervated through cranial nerve XI. However, a recent study showed that this muscle is actually supplied by rostral spinal motor neurons that, unusually, ascend to exit through cranial nerve XI [51]. Hence, our data shows that brainstem motor neurons are, in general, particularly resistant to aging and ALS.

The other notable outlier is the external anal sphincter. The preservation of synapses in this muscle may reflect unusual properties. The EAS is a rhabdosphincter – a specialized muscle sphincter composed of striated skeletal muscle – innervated by neurons that reside in the Onuf's nucleus, a distinct nucleus of neurons in the ventral horn of the sacral spinal cord. Although these neurons appear morphologically similar to alpha motor neurons and innervate striated muscle which is under voluntary control, the identity of these neurons as being somatic or autonomic in nature remains under debate [52], [53]. For example, motor neurons in Onuf's nucleus are severely affected in Shy Drager Syndrome [41], a disease that causes the degeneration of autonomic neurons in the brain. Therefore, perhaps these neurons are protected in aging and ALS because their genetic profile more closely resemble neurons in the brain than spinal motor neurons. With these findings in mind, an important future direction is to identify the factors that protect cranial motor neurons from aging and disease.

### Molecular differences between afflicted and spared NMJs

The similar patterns of structural change in NMJs of old and SOD-G93A mice suggested that molecular parallels might also exist. To test this possibility, we focused on two proteins that have been implicated in ALS: CRMP-4a and TDP-43. Upregulation and mis-localization of CRMP4a and TDP-43, respectively, have been documented in motor neurons. Furthermore, mutations in TDP-43 lead to ALS and frontotemporal dementia of SOD-G93A mice [44]. We found that CRMP4a is also upregulated and TDP-43 mislocalized in motor neurons of old mice that supply susceptible muscles. In contrast, motor neurons with spared NMJs maintain the normal expression level and distribution of these two molecules, both in old mice and in SOD-G93A mice. These results, therefore, provide evidence that NMJs, in aging and ALS, share molecular as well as structural features.

## Materials and Methods

### Source of mice

Thy1-XFP transgenic mice were described previously [23]. They were bred and aged in our colony. All motor axons are labeled in line YFP-16 and small subsets of motor axons are labeled in line YFP-H. We also obtained young (4 to 5 month-old) and aged (22 to 28-month-old) C57BL/6 mice from the National Institute of Aging and SOD-G93A transgenic mice from the Jackson Laboratory. All experiments were carried out under NIH guidelines and an animal protocol approved by Harvard University Animal Studies Committee.

### Immunostaining

Mice were anesthetized with sodium pentobarbital and perfused transcardially with 4% p-formaldehyde in 0.1 M phosphate-buffered saline (PBS; pH 7.4). Muscles were then dissected, post-fixed for 30 min and blocked overnight at 4°C (1% Triton X-100, 4% BSA in PBS). They were then incubated for 1–3 days with neurofilament (1∶500) and synaptotagmin-2 (1∶250) antibodies, washed with PBS for 3 hours and incubated for 24 hours with Alexa 555-α-bungarotoxin (Molecular Probes, Eugene, OR) together with secondary antibodies (Alexa-488 anti-mouse IgG1 and Alexa-647 anti-mouse IgG2A; Molecular Probes). After washing for 3 hours in PBS, muscles were whole-mounted on slides in Vectashield (Vector Labs).

In some cases, muscles were incubated for 2 hr with 5 µg/ml Alexa 555-conjugated α -bungarotoxin following post-fixation and before further processing. To stain muscle sections, muscles were dissected and immersed in 30% sucrose overnight. 30 to 40 µm longitudinal sections were obtained and stained as described above.

Muscle fiber composition was determined by immunostaining 10 µm cross-sections of unfixed muscles. Sections were blocked for 30 minutes at room temperature (0.1% Triton X-100, 4% BSA in PBS). They were immediately incubated with anti-MyHC I [A4840 from Developmental Studies Hybridoma Bank (DSHB) and NCLslow from Leica Microsystems/Novacastra Laboratories], anti-MyHC IIA (2F7 and SC-71 from DSHB), or anti-MyHC IIB (BFF3 from DSHB) for 2 hours. Sections were washed 3 times for 5 minutes and immediately incubated with alexa-conjugated secondary antibodies. After a final wash, sections were mounted and visualized.

Spinal cords and brainstem were dissected, postfixed for 2 hours and immersed in 30% sucrose overnight. 40 µm cryosections were obtained, blocked for 1 hour at room temperature (0.2% Triton X-100, 4% BSA in PBS). They were then stained with antibodies against CRMP4a or TDP-43 in blocking solution overnight, washed 3 times with PBS and stained with alexa-conjugated secondary antibodies. All sections were also stained with Neurotrace to visualize neuronal cell bodies. Sources of antibodies were as follows: anti-neurofilament (smi-312, Covance); anti-synaptotagmin 2 (znp-1), Zebrafish International Resource Center (Eugene, OR); anit-CRMP4a (ab23951, Abcam), anti-TDP-43 (3449S, Cell Signaling).

### Histological analysis

Maximum intensity projections of confocal stacks obtained from aged (≥24 months) and young adult (2–6 months) junctions were created using Metamorph 6.03 software (Molecular Devices, Sunnyvale, CA). Innervation of AChRs was defined as co-localization of axon terminals (green) with fluorescently labeled AChRs (red). Synaptic occupancy was delineated using Metamorph from the overlapping area between the terminal axon and receptors. Synaptic defects were scored using the definitions in Ref. 19: (a) NMJs were scored as fragmented if they contained 5 or more AChR island and/or a segment of the postsynapse showing severe abnormalities such as small and/or irregularly shaped AChR clusters. (b) Faint AChR clusters were defined as noticeably dimmer postsynaptic sites compared to those in the same confocal plane. (c,d) Full or partial denervated postsynaptic sites were fully or completely unapposed by nerve terminals, represented by YFP or a presynaptic marker. (e) Sprouts were terminal extensions of the nerve >1 µm beyond the border of AChR cluster in any direction. (f) Axonal swellings were anomalous distensions of preterminal portions of the axon. (g) Axonal atrophy was characterized by thinning of the axon by approximately 2 µm compared to the diameter of normal axons. (h) Multiple innervation was scored when two or more preterminal axons were seen to enter a single postsynaptic site. In all cases, we sampled NMJs throughout all regions of a muscle, generally by capturing a set of 20× or 40× images centered on the end-plate band at each of three levels through the z-axis.

To determine motor unit size, entire motor axons and their NMJs were imaged at high resolution as above. Z stacks were flattened (Metamorph) and reconstructions generated using Photoshop CS. The branch order for each motor axon was determined by constructing a complete branching diagram for the entire motor axon as described previously [32].

## Supporting Information

Figure S1 **Fragmentation of neuromuscular junctions in young adult extraocular muscles.** Extraocular muscles from young adult transgenic mice that expressed YFP in axons (green) were stained with BTX to label AChRs (Red). In young EOMs, AChR are often highly fragmented compared to AChR clusters in other muscles (see Figure 1A for a comparison to a young EDL NMJ). Scale bar = 20 µm.
(TIF)

Figure S2 **Neuromuscular junctions in young adult and old muscles.** Longitudinal sections from transgenic mice that expressed YFP in axons (green) were stained with BTX to label AChRs in the postsynaptic membrane (red). A) Young adult sternomastoid. B) Old sternomastoid. C) Young adult frontalis. D) Old frontalis. Scale bar = 10 µm.
(TIF)

Figure S3 **Synapses are differentially affected within an aged motor unit.** The figure shows 46 NMJs that comprise one axon's motor unit in a 2 year old mouse. As shown by the color code (bottom, right corner), some junctions are completely innervated (red circles) while the AChRs at other junctions are only partially covered by nerve terminals (purple, yellow, green and blue circles). Numbers in left upper corners represent the junction identity used for branch analysis in Figure 9.
(TIF)
