# Supplementary figures and images for: Shared Resistance to Aging and ALS in Neuromuscular Junctions of Specific Muscles
Source: PLoS One. 2012 Apr 2;7(4):e34640. doi: 10.1371/journal.pone.0034640 (PMC3317643; doi:10.1371/journal.pone.0034640)

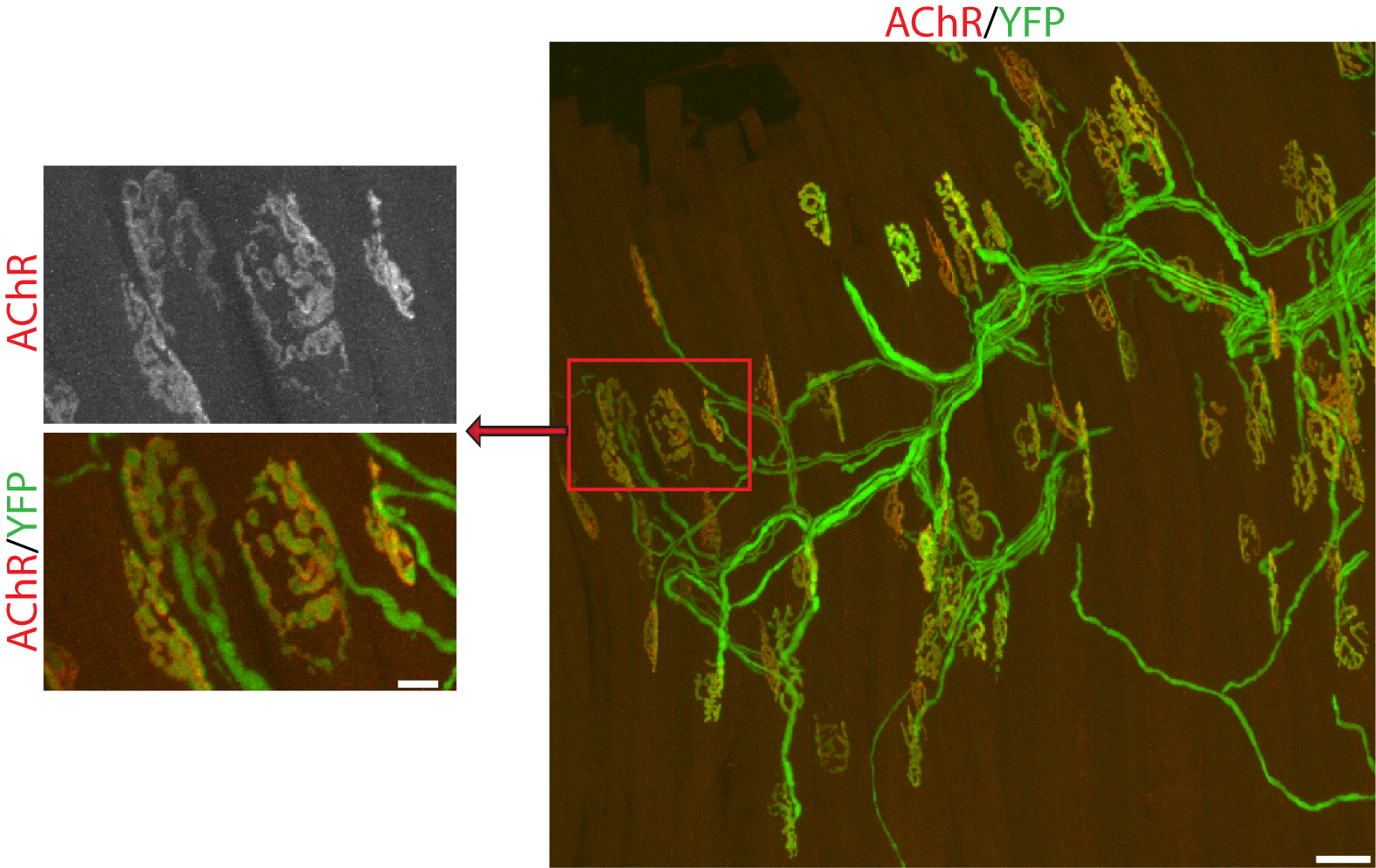

Supplement: Figure S1 — Fragmentation of neuromuscular junctions in young adult extraocular muscles. Extraocular muscles from young adult transgenic mice that expressed YFP in axons (green) were stained with BTX to label AChRs (Red). In young EOMs, AChR are often highly fragmented compared to AChR clusters in other muscles (see Figure 1A for a comparison to a young EDL NMJ). Scale bar = 20 µm. (TIF) [file pone.0034640.s001.tif]

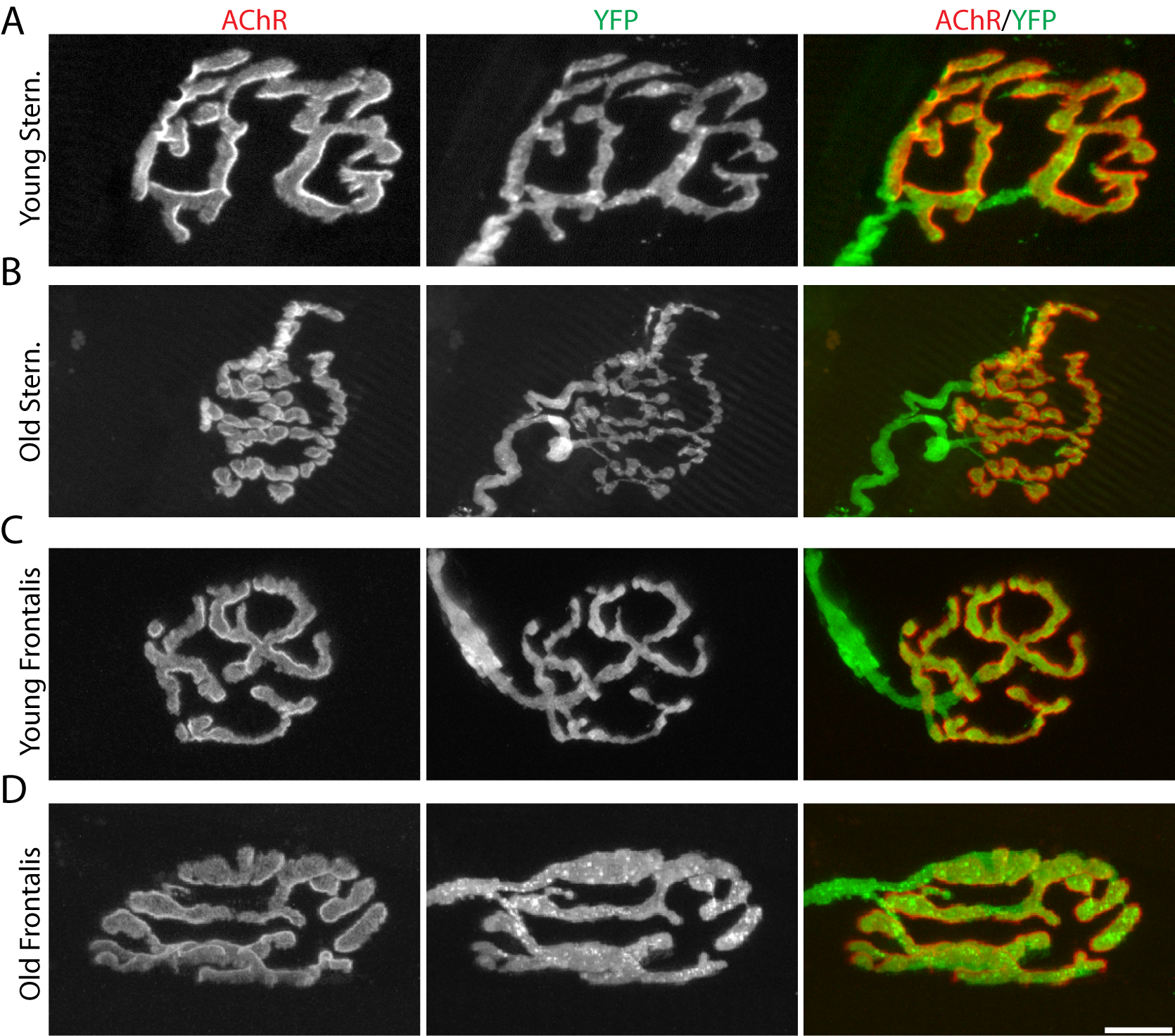

Supplement: Figure S2 — Neuromuscular junctions in young adult and old muscles. Longitudinal sections from transgenic mice that expressed YFP in axons (green) were stained with BTX to label AChRs in the postsynaptic membrane (red). A) Young adult sternomastoid. B) Old sternomastoid. C) Young adult frontalis. D) Old frontalis. Scale bar = 10 µm. (TIF) [file pone.0034640.s002.tif]

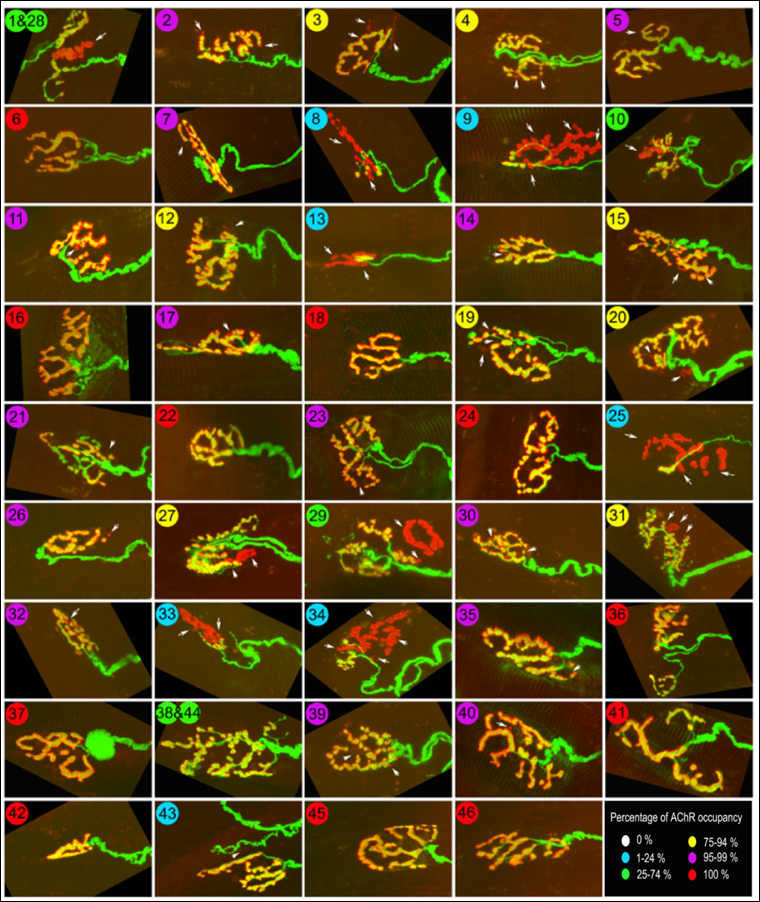

Supplement: Figure S3 — Synapses are differentially affected within an aged motor unit. The figure shows 46 NMJs that comprise one axon's motor unit in a 2 year old mouse. As shown by the color code (bottom, right corner), some junctions are completely innervated (red circles) while the AChRs at other junctions are only partially covered by nerve terminals (purple, yellow, green and blue circles). Numbers in left upper corners represent the junction identity used for branch analysis in Figure 9. (TIF) [file pone.0034640.s003.tif]
